# A Syriac Medical *Kunnāšā* of Īšōʿ bar ʿAlī (9th c.): First Soundings*

**DOI:** 10.1163/2212943x-00503015

**Published:** 2017-01

**Authors:** Grigory Kessel

**Affiliations:** Austrian Academy of Sciences and University of Manchester

**Keywords:** Syriac medicine, medical handbooks, ʿĪsā b. ʿAlī, Paul of Aegina, Syriac translations

## Abstract

A little-known thirteenth-century manuscript preserved in Damascus contains by far the largest Syriac medical work that has survived till today. Despite the missing beginning, a preliminary study of the text allows us to argue that it is the medical handbook (entitled *Kunnāšā*) of Īšōʿ bar ʿAlī, a ninth-century physician and student of Ḥunayn b. Isḥāq. The seven books of the handbook appear to follow the model of Paul of Aegina’s *Pragmateia* both in composition and content. The actual significance of the handbook in the history of Syriac and Arabic medicine is yet to be assessed, but there can be no doubt that it will be a pivotal source that illustrates the development of Syriac medicine during a period of four centuries at the moment when it was being translated to lay the foundations of the nascent medical tradition in Arabic.

## 1 Manuscript

The unique manuscript preserved in the library of the Syriac Orthodox Patriarchate near Damascus is not totally unknown but unfortunately it has never received a proper description, and due to its inaccessibility it remained out of scholarly attention and inquiry.^1^ At present, codex Syrian Orthodox Patriarchate 238 (hereafter sop 238, a previous shelfmark was 6/1) consists of 439 folios, but only the first 435 of those belong to the original codex whereas the four last ones were added later. What we find on those supplementary folios (sop 238, ff. 436–440) seems to be a fragment from an independent Syriac medical manuscript of a later date. The text there is divided into sections that deal with anatomy, the preparation of theriac “according to the opinion of Galen” and “Indian drugs”.^2^ The scribe mentions that he translated the text from Arabic.

The original codex is damaged and lacks some folios. Especially noticeable is the absence of the opening part (the text begins in the middle of the second quire) although five folios (sop 238, ff. 431–435) from the missing part can now be found attached to the end of the text. Those five folios contain the table of contents (damaged) as well as the very beginning of the original text (first chapter of Mēmrā i) and a page from the introduction where the author traces the history of the art of medicine and healing back to Asclepius.

According to the colophon (sop 238, f. 430^v^) that follows the text, the manuscript was completed on September 30, ag 1535 [= 1224 ce] by deacon Basil the son of Rabban Yoḥannan, an archpriest of Melitene.^3^ The manuscript was copied in Mosul at the time when the scribe was disciple of “the archiatros of the East,” Rabban Abu Saʿid.^4^ The scribe of the manuscript, Basil son of Yoḥannan from Melitene, is known to have copied in 1221 ce an additional important Syriac manuscript with works on secular subjects that has survived in modern apographs.^5^ Furthermore, there is an intriguing possibility to identify the scribe with a scribe Basil Meliteniotes, a Greek Orthodox Armenian, who produced in 1226 ce a famous illustrated Greek manuscript preserved in the Gennadius Library (Athens).^6^

The medical text is written on paper in a very condensed ductus of Serto which makes reading the manuscript a serious challenge despite its overall good condition. The manuscript contains a large number of notes left by its readers that can shed light on its history. Particularly, the notes on ff. 402^v^–403^r^ were penned by the ecclesiastical leaders of the Syrian Orthodox Church; perhaps the two most well-known names are Masʿūd of Zāz, a patriarch of Ṭūr ʿAbdīn (1431–1512) and Severus, a metropolitan of Syria and the future patriarch Ignatius Aphrem Barsoum (1887–1957).

Finally, the presence of the Armenian quire signatures and Arabic glosses hint at wide circulation of the manuscript.

## 2 Text

### 2.1 Title

Due to the loss of the beginning of the manuscript we are bereft of priceless information about the title of the work and its author. Nevertheless, while going through the text one’s attention is regularly drawn to the term *Kunnāšā*, as it features, for example, in the opening and closing rubrics of some of the Memrē. Considering that the term *Kunnāšā* was regularly applied to medical handbooks, it is natural to see it as the title of the magnificent work before us.

Moreover, we cannot exclude the possibility that an extended version of that title featured in the work’s original title, that could be, following the practice of that time, quite verbose. For instance, we find at the end of Mēmrā iv (sop 238, f. 304^v^):

ܟܘܢܫܐ ܕܥܠ ܥ̈ܠܬܐ ܕܟܘܪ̈ܗܢܐ ܘܫܘܘܕܥܝ̈ܗܘܢ ܘܐܣܝܘܬܗܘܢ

*Kunnāšā* on the causes of diseases, their symptoms and treatment.

### 2.2 Composition and Content

The text of the *Kunnāšā* consists of seven Memrē that are further subdivided into chapters. Below I provide a concise summary of their contents.

Mēmrā i (sop 238, ff. 1^r^–92^r^). 106 chapters. Hygiene, Diet, *Materia Medica*

Complaints of pregnant women, regimen (ܕܘܒܪܐ) of children, middle-aged, and old people, air, water, wine, regimen fitting to the different seasons, fatigue, purgation, bath (ܡܣܚܘܬܐ), food, *materia medica*, simples, purgative drugs, classification of the simples, drugs not mentioned by Dioscorides and Galen (some of those are explicitly marked as Persian and Indian), rules for the preparation of compounds.

Mēmrā ii (sop 238, ff. 92^v^–151^v^). 47 Chapters. Local Ailments—Head

Head sicknesses caused by overheating, supercooling, drunkenness, concussion, fall; brain disorders, torpor (ܩܘܡܐܛܘܣ), phrenitis (ܦܪܗܢܝܛܝܣ), madness (ܡܐܢܝܐ), delirium (ܨܒܪܐ), lethargy (ܠܝܬܐܪܓܘܣ), epilepsy (ܗܦܝܠܝܡܦܣܝܐ), insanity (ܫܛܘܪܘܬܐ), apoplexy (ܐܦܘܦܠܝܟܣܝܐ), loss of memory, melancholy (ܡܗܠܐܢܟܘܠܝܐ), spasm (ܡܩܝܣܘܬܐ), numbness (ܬܢܘܒܘܬܐ), trembling (ܪܥܠܐ), afflictions of the hair, alopecia (ܬܥܠܘܬܐ), afflictions of the skin, lice (ܩܠܡ̈ܐ), nits (ܢܒ̈ܐ), love-sick people.

Mēmrā iii (sop 238, ff. 152^r^–211^r^). 81 Chapters. Local Ailments—Eyes, Ears, Chest

Pus that forms in the eyes (ܛܦܪ̈ܐ ܕܒܥܝ̈ܢܐ), ophthalmia (ܕܓܢܐ), eye swelling (‮ܢܦܝܚܘܬܐ ܒܥܝ̈ܢܐ‬‎), itching in the eyes (ܚܟܬܐ ܕܒܥܝ̈ܢܐ), inflammation in the eyes (ܦܠܗܓܡܘܢܝ), eyelids (ܬܠܝ̈ܦܐ) disorders; diseases of sclera (ܟܘܬܝܢܐ), ears, nostrils (ܢܚܝܪ̈ܐ), teeth, tongue, mouth, chest; warts (ܕܩܠܘ̈ܢܐ), cough (ܫܥܠܐ), inflammation of the lungs (ܦܗܪܝܦܠܡܘܢܝܐ), spitting blood (ܪܩܩ ܕܡܐ), wasting (ܦܬܝܣܝܣ), pleurisy (ܕܩܪܬܐ), heart diseases, swoon (ܥܦܬܐ), humpback (ܟܘܣܬܢܘܬܐ).

Mēmrā iv (sop 238, ff. 211^v^–304^v^). 67 Chapters. Local Ailments—Abdomen, Genitals

On stomach diseases (ܥܠ ܚ̈ܫܐ ܕܡܬܩܝܡܝܢ ܒܐܣܛܘܡܟܐ), bulimia (ܒܘܠܝܡܘܣ), vomiting (ܬܝܘܒܐ), hiccups (ܦܘܟܬܐ), cholera (ܟܘܠܗܪܐ), jaundice (ܝܪܩܢܐ), diarrhoea (ܕܝܠܐ), stomach spasm (ܡܥܣܐ), dysentery (ܕܘܣܢܛܪܝܐ), intestinal obstruction (ܐܝܠܗܐܘܣ), worms (ܫܘܫ̈ܠܐ); diseases of the kidneys (ܟܘܠܝ̈ܬܐ), bladder (ܫܠܦܘܚܬܐ), genitals (ܓܒܪܘܬܐ), flow of semen (ܕܘܒܐ ܕܙܪܥܐ), sexual desire, disorders of menstruation (ܟܣܦܐ), gout (ܦܘܕܐܓܪܐ), diseases of the testicles (ܐܫ̈ܟܐ), haemorrhoids (ܣܘܪ̈ܓܐ).

Mēmrā v (sop 238, ff. 305^r^–358^v^). Some 90 Chapters.^7^ Fevers, External Ailments, Prognostication

Fevers, hectic (ܐܩܛܝܩܘܣ), synuchos (‮ܣܘܢܘܟܘܣ‬‎), skin inflammation (‮ܡܫܪܐ‬‎), pustules (‮ܚܡܛܐ‬‎), tertian fever (‮ܛܪܝܛܘܣ‬‎), quartan fever (‮ܛܐܛܐܪܛܗܘܣ‬‎), semitertian fever (‮ܗܡܝܛܪܝܘܣ‬‎), compound fevers (‮ܐܫܬܘ̈ܬܐ ܡܪ̈ܟܒܬܐ‬‎), ailment of ulcers (‮ܫܘܚܢܐ‬‎), itching (‮ܚܟܟܐ‬‎), cancer (‮ܣܪܛܢܐ‬‎), hernia (‮ܬܠܚܐ‬‎), hardened swelling (‮ܛܪܢܘܬܐ‬‎), tumour (‮ܪܘܒܠܐ‬‎), tetter (‮ܚܙܙܝܬܐ‬‎), leprosy (‮ܐܪܝܢܘܬܐ‬‎); different forms of prognostication, including urine (‮ܬܦܫܘܪܬܐ‬‎).

Mēmrā vi (sop 238, ff. 358^r^–361^r^). [In 94 Chapters]. Lethal Poisons (‮ܣܡܡ̈ܢܐ ܩܛܘ̈ܠܐ‬‎)

Although in its actual form the Mēmrā briefly describes just a limited number of recipes^8^ against, among others, the stings of a bee, night bat and scorpion, in its original form it was much more substantial. As we can learn from the surviving part of the index to that Mēmrā (covers only chapters 68–94) it dealt with antidotes against the bites and stings of various insects and animals (ranging from the expected, such as snakes, to such unusual cases as crocodiles).

Mēmrā vii (sop 238, ff. 361^v^–431^v^). 25 Chapters. Compound Drugs, Surgery

The final Mēmrā is not homogeneous. Thus it begins with a discussion of the rules for producing compound drugs, then moves to recipes for theriac, antidotes (‮ܐܢܛܝܕܘܛܘܣ‬‎) and hiera (‮ܐܝܗܪܐ‬‎) and pays much attention to the preparation of innumerable sorts of pills (‮ܟܟ̈ܠܬܐ‬‎), gargles (‮ܥܘܪܥܪܐ‬‎), bandages (‮ܥܨܒܐ‬‎), compresses (‮ܐܣܦܠܢܝܐ‬‎), elixirs (‮ܟܣܝܪܝܢ‬‎) and collyria (‮ܫܝ̈ܦܐ‬‎). Special chapters are devoted to the ailments of the teeth and eyes. The Mēmrā ends with two auxiliary chapters on measures and substitute drugs.

Both in its composition and content the *Kunnāšā* reminds us of the medical compendia and handbooks that are known from the late antique and early Islamic periods.^9^ The *Kunnāšā* covers all the standard subjects of the medical handbooks: regimen and *materia medica* (Mēmrā i), diseases pertaining to a particular body part are presented by and large following the principle from head to toe (Memrē ii, iii and iv), fevers and external diseases (Mēmrā v), poisons (Mēmrā vi), compound drugs (Mēmrā vii). What strikes one perhaps most of all is the author’s splendid command of pharmacology (notably, Mēmrā i is the largest) for the treatment of each diseases is replete with various recipes.

However, among the available manuals the *Pragmateia* of Paul of Aegina (7th c.) is most likely to have been the model of this *Kunnāšā*. Indeed, at first glance the two works appear to have much in common. For example, each of the two handbooks is divided into seven parts that begin with complaints of pregnant women and end with measures and substitute drugs; each chapter first presents the aetiology and symptoms of an ailment and afterwards its treatment. Nevertheless, close comparison demonstrates also considerable differences. Thus, whilst Paul’s Book Three (local diseases) relates to Memrē ii, iii and iv, the other Memrē usually cover the material from more than one Book (for example, Mēmrā i covers the material present in Books One and Seven and Mēmrā v merges the subjects from Books Two and Four). Furthermore, whereas some material of Paul’s *Pragmateia* is not present whatsoever (for example, therapy of children), in other subjects the *Kunnāšā* demonstrates a higher level of detail and proficiency (see for instance the chapters on ophthalmology in Mēmrā iii). It is therefore may well be that the text of Paul’s *Pragmateia* was mediated through another source. The relationship between Paul’s compendium and the *Kunnāšā* merits further research because, as we shall see below, the influence of the *Pragmateia* can be traced not only on the level of composition but also in content.

### 2.3 Sources

The names of medical authorities are sparingly scattered in the text of the *Kunnāšā*. Thus we come across references and citations attributed to Hippocrates, Dioscorides, Rufus of Ephesus, Galen, Philagrius, Oribasius, Aetius of Amida, Alexander of Tralles, and Paul of Aegina. Some names escape straightforward identification, for example Theodoretus and Theophilus. As for the native Syriac authors, only two names have been noticed, Ḥunayn b. Isḥāq and Abū Zakarīyāʾ Yūḥannā b. Māsawaih. It goes without saying that the explicit indications are nothing more than just the tip of the iceberg and it should be possible to detect the principle sources of the *Kunnāšā*. That issue will undoubtedly be one of the central questions on the agenda for further research. Nevertheless, already based on a brief acquaintance with the text it is possible to argue that Paul of Aegina occupies a very prominent position. A reading of selected chapters of the *Kunnāšā* shows the wide-ranging influence of Paul’s *Pragmateia*.

By way of example, let me provide a citation from the beginning of a seven-page long chapter on melancholy (Mēmrā ii.26, sop 238, ff. 124^v^–128^r^) that, as we shall see shortly, is a faithful version of the opening section on melancholy in Paul’s *Pragmateia*.

**Paul. Aeg. 3.14.1 (i. 156.9–15 Heiberg)**^10^

[1] Ἡ μελαγχολία παραφροϲύνη τίϲ ἐϲτιν ἄνευ πυρετοῦ [2] ἐπὶ μελαγχολικῷ μάλιϲτα χυμῷ γινομένη κατειληφότι τὴν διάνοιαν, [3] ποτὲ μὲν αὐτοῦ πρωτοπαθοῦντοϲ τοῦ ἐγκεφάλου, [4] ποτὲ δὲ τῷ ὅλῳ ϲυμμεταβαλλομένου ϲώματι· [5] καὶ τρίτον δὲ μελαγχολίαϲ εἶδόϲ ἐϲτιν, ὃ φυϲῶδέϲ τε καὶ ὑποχονδριακὸν καλοῦϲιν, [6] ἐπὶ φλεγμονῇ τῶν περὶ τὸν ϲτόμαχον ὑποχονδρίων ϲυνιϲτάμενον [7] ποτὲ μὲν αὔραϲ τινὰϲ μοχθηράϲ, [8] ποτὲ δὲ καὶ τῆϲ οὐϲίαϲ τοῦ χυμοῦ μέροϲ ἀναπεμπόντων πρὸϲ τὸν ἐγκέφαλον.
[1] Melancholy is a disorder of the intellect without fever, [2] occasioned mostly by a melancholic humour seizing the understanding; [3] sometimes the brain being primarily affected, [4] and sometimes it being altered together with the entire of the body. [5] And there is a third type called the flatulent and hypochondriac, [6] occasioned by inflammation of one of the parts in the hypochondria adjoining to the stomach, [7] by which sometimes noxious vapours, [8] and sometimes a part of the substance of the humour, is transmitted to the brain.

*Kunnāšā*, Mēmrā ii.26 (sop 238, f. 124^v^)^11^

܆[1] ܡܗܠܐܢܟܘܠܝܐ ܗܟܝܠ ܐܝܬܝܗ̇ ܆ ܢܟܝܢܐ ܕܡܘܚܐ ܕܪܫܐ ܆ ܕܠܗ ܢ̇ܦܩ ܚܘܒܠܐ ܕܚܘܫܒܐ ܘܐܒܝܕܘܬ ܗܘܢܐ . ܕܒܠܥܕ ܐܫܬܐ ܗ̇ܘܝܐ ܇ [2] ‭‬ ܡ̣ܢ ܟܘܡܘܣ ܡܗܠܐܢܟܘܠܝܩܝܐ ܕܠ̇ܒܟ ܠܬܪܥܝܬܐ . [3] ‭‬ ܟܕ ܒܙܒܢ ܡ̇ܢ ܗܘ ܡܘܚܐ ܩܕܝܡܘܬ ܚܫܘܫܘܬܐ ܢܚܫ . [4] ‭‬ ܒܙܒܢ ܕܝܢ ܟܕ ܥܡ ܟܠܗ ܦܓܪܐ ܆ ܡܐ ܕܝܬܝܪ ܥܠܘܒ‭‬^12^ ܟܘܡܘܣ ܗܢܐ . ‭‬ [5] ܘܬܘܒ ܐܕܫܐ ܬܠܝܬܝܐ ܗ̇ܘܐ ܇ ܗ̇ܘ ܕܡ̣ܢ ܓܘܐ ܡܬܩܪܐ ܡܢܦܚܐ ܘܡܪܩܩܢܐ ܆ [6] ‭‬ ܕܡ̣ܢ ܦܠܗܓܡܘܢܝ ܕܚܕ ܡ̣ܢ ܗܕ̈ܡܐ ܕܨܝܕ ܐܣܛܘܡܟܐ ܐܘ ܡܪܩܩܐ ܡܬܩܝܡ ܆ [7] ‭‬ ܐܘ ܡ̣ܢ ܟܘܡܘܣ ܥܒܝܐ ܘܦܠܓܡܢܐ ܟܕ ܢܫ̈ܒܐ ܒܝ̈ܫܐ ܓܕܐ ܡ̇ܣܩ ܠܘܬ ܡܘܚܐ ܆ [8] ‭‬ ܒܙܒܢ ܕܝܢ ܆ ܘܐܦ ܟܕ ܡܢܬܐ ܡܢܗ ܕܟܘܡܘܣ ܬܣܩ.܆

[1] Melancholy is a disorder of the brain *accompanied by an injury of the intellect and insanity* without fever, [2] occasioned by a melancholic humour seizing the understanding; [3] sometimes the brain being primarily affected, [4] and sometimes it being altered together with the entire body *when that humour abundantly exceeds*. [5] And there is a third type called the flatulent and hypochondriac,^13^ [6] occasioned by inflammation of one of the parts near the stomach or hypochondria, [7] by which sometimes noxious vapours *are secreted from thick and phlegmatic humour* and are transmitted to the brain, [8] and sometimes a part of the substance of the humour ascends.

A comparison of the original Greek with the version found in the *Kunnāšā* demonstrates that its author basically reproduced the key passage from Paul of Aegina’s discussion of melancholy while providing it with just a few additions that were apparently necessary to ensure the correct interpretation of the text. We should not exclude, however, a possibility that both the text of Paul’s treatise and the interpolations might go back to an intermediary source that is of course already lost. Nevertheless, a comprehensive study of *Kunnāšā* will help to clarify the issue. A comparison with other relevant sources, such as the *Small Compendium* in seven books of Yūḥannā b. Sarābiyūn, may also shed light on the background and sources of *Kunnāšā* (a chapter on melancholy in the *Small Compendium* differs from its treatment in *Kunnāšā*).^14^

Thus paragraph [1] enumerates other possible effects that may coincide with a disorder of the brain. The second type of melancholy [4] may embrace the entire body, and for that reason the Syriac author elucidates the point by stressing that such a condition is possible “when that humour abundantly exceeds.” And finally a description of the third type [7] is provided with the helpful precision that behind the inflamed hypochondria that produces the vapours stands a “thick and phlegmatic humour.”

As demonstrated by Peter E. Pormann, the *Pragmateia* of Paul of Aegina was translated into Syriac in the eighth or ninth century and the Syriac version was used by at least two Syriac authors, Yūḥannā b. Sarābiyūn (9th c.) and Bar Bahlūl (10th c.).^15^ As far as availability of the Syriac text is concerned, only a limited number of brief citations are preserved in the *Lexicon* of Bar Bahlūl. For that reason the text of the *Kunnāšā* will contribute greatly to our better awareness of the Syriac version of the *Pragmateia* and will help to comprehend its significance for the Syriac medical tradition.^16^

As mentioned earlier, the *Kunnāšā* contains a large number of quotations from Greek sources. Since the majority of those translations have vanished for good, it can deservedly be considered an invaluable treasure chest for research into the Syriac translations of Classical medical literature. Particularly, it is of utmost importance for the ninth-century translations that, as it seems, were readily available to Īšōʿ bar ʿAlī. By way of example, it is worth analyzing a quotation from Hippocrates’ *Aphorisms*, the Syriac version of which happens to survive.

**Aph. ii.47 (iv. 482.17–18 Littré)**

Περὶ τὰϲ γενέσιαϲ τοῦ πύου οἱ πόνοι καὶ οἱ πυρετοὶ ξυμβαίνουσι μᾶλλον, ἢ γενομένου.
Pains and fevers occur rather at the formation of pus than when it is already formed.

*Kunnāšā*, Mēmrā v (sop 238, f. 331^v^)

܆ܐܦ ܐܝܦܦܘܩܪܐܛܝܣ ܗܟܢܐ ܕܒܡܘܠܕܐ ܠܡ ܕܡܘ ܓܠܐ ܓܕܫܝܢ ܟܐܒ̈ܐ ܘܐܫܬܘ̈ܬܐ ܝܬܝܪ ܡܢ ܡܐ ܕܐܬܝܠܕ.܆A1܆

And also Hippocrates: Pains and fevers occur rather at the generation of pus than when it is already generated.

An anonymous Syriac translation (Pognon, *Une version syriaque*, p. 9 lines 1–2):

܆ܒܗܘܝܐ ܕܡܘܓܠܐ ܟܐܒ̈ܐ ܘܐܫܬܘ̈ܬܐ ܝܬܝܪܓܕܫܝܢ ܡ̣ܢ ܡܐ ܕܟܕ ܗ̣ܘܐ.܆A2܆

Pains and fevers occur rather at the production of pus than when it is already produced.

The two versions are so close to each other that the minimal difference can hardly be conveyed in translation and yet from the point of view of Syriac they seem to display different dispositions and approaches in translation techniques. Thus, whereas in A1 two derivatives of the verb γίγνομαι (γένεσιϲ and γενόμενος) are rendered employing Syriac verb yld, in A2 another synonymous word was used, hwa. The choice made in A1 is closer semantically to the original Greek, whilst A2 opts for linguistic precision and employs a calque. Another contrast between two versions can be observed in the position of the predicate ܓܕܫܝܢ. In line with the previous point, A1 demonstrates a preference to produce a rendering closer to standard Syriac, whereas A2 exhibits a predilection towards a more literal rendering of the original.

As far as we know, Īšōʿ bar ʿAlī was not active as a translator from Greek^17^ and for that reason we should assign differences between two translations not to his personal input but rather to the version available to him. If this is indeed the case, he must have had at his disposal the translation of the *Aphorisms* made by his master Ḥunayn b. Isḥāq. Intriguingly, such a conclusion urges the reappraisal of a widely accepted assertion of Rainer Degen, who argued that the translation edited by Pognon should be attributed to Ḥunayn.^18^ That small example demonstrates the *Kunnāšā*’s potential significance for the study of the Syriac translations of the *Aphorisms*, an area of inquiry that has generated a stream of publications in recent years.^19^

Besides the written medical works that establish the general framework of the *Kunnāšā* one should not overlook immediate input by its author, who regularly adds his own opinion after presenting the (probably borrowed) convictions of others. Since that characteristic appears most often in the discussion of disease treatments, we should attribute such personal remarks and comments to the author’s first-hand experience as a practitioner.

## 3 The Author

As mentioned earlier, the unique manuscript of the *Kunnāšā* is damaged and lacks the very beginning of the treatise that must have contained an indication of both the author and the title. However, we are fortunate to have a few pages from that opening part of the treatise that were bound at the end of the treatise. Besides the table of contents, we find there also a small fragment from the introduction to the *Kunnāšā* that ends with the following rubric (sop 238, f. 435^r^):

ܫܠܡ ܡܦܩܒܪܘܚܐ ܕܪܒܢ ܝܫܘܥ ܒܪ ܥܠܝ ܐܣܝܐ ܡܝܬܪܐ ܫ̇ܘܐ ܠܩܘ̈ܠܣܐ

The introduction of Rabban Īšōʿ bar ʿAlī, an excellent physician who is worthy of praise, is completed.

The expression *mappaq b-rūḥā* (“introduction, preface”) indicates a special type of introduction that corresponds to the Greek *prooemium* and that was common in Syriac scholarly literature.^20^ Relying on that rubric, the *Kunnāšā* should be attributed to Īšōʿ bar ʿAlī or in Arabic ʿĪsā b. ʿAlī, who is known as a personal physician of Caliph al-Muʿtamid (r. 870–892), a disciple of Ḥunayn b. Isḥāq and the author of the Syriac-Arabic *Lexicon* that is extant in multiple manuscript copies.^21^ The confusion between three known authors with similar names has been recently clarified by Aaron Butts, who cogently argued that Īšōʿ bar ʿAlī, the author of the *Lexicon*, has to be identified “with physician ʿĪsā b. ʿAlī, who lived in the second half of the ninth century and was a student of Ḥunayn b. Isḥāq.”^22^ That line of argumentation has the immediate corollary that the ninth-century ʿĪsā b. ʿAlī, a disciple of Ḥunayn, must be divorced from the tenth-century ʿĪsā b. ʿAlī al-Kaḥḥal (d. ca. 1010), a disciple of Abū l-Faraǧ ʿAbd Allāh b. al-Ṭayyib and the author of an influential ophthalmological treatise.^23^

If this is indeed so, one wonders if there is any positive evidence of the medical works of Īšōʿ bar ʿAlī. Unfortunately, despite recurrent mention in the Syriac sources of Īšōʿ bar ʿAlī as physician (ܐܣܝܐ), apart for a fragment (on which see below) none of his works in Syriac is extant.^24^ As for the Arabic tradition, Ibn Abī Uṣaibiʿa (d. 1270) in his *ʿUyūn al-anbāʾ fī ṭabaqāt al-aṭibbāʾ*, while extolling ʿĪsā b. ʿAlī’s excellence in the science of medicine, mentions that he produced many works (*taṣānīf*) among which two are particularly mentioned, the *Book of the benefits that can be obtained from the parts of animals* (*Kitāb al-Manāfiʿ allatī tustafādu min aʿḍāʾ al-ḥayawān*) and the *Book of Poisons* (*Kitāb al-Sumūm*) in two parts (*māqālatān*). Whereas the former work is extant but remains so far unpublished,^25^ the latter one is lost except for a handful of citations in later sources.^26^

With all that information in hand, is there a chance to establish a connection between the *Kunnāšā* and the literary output of Īšōʿ bar ʿAlī? It seems that there is one point of pivotal significance. Namely, Ibn Abī Uṣaibiʿa records that Īšōʿ bar ʿAlī was the author of a *Book of Poisons* that was composed in two parts. As we have seen, the *Kunnāšā* contains two Memrē that deal with poisons. It is not the Memrē themselves that are important now, though, but an introductory rubric that opens Mēmrā vi. In it the scribe apologizes for reproducing the original Mēmrā only partially because “the author composed two other Memrē about poisons” (sop 238, f. 358^v^). It is thus very tempting to see in those “two Memrē” on the subject of poison a treatise that is recorded by Ibn Abī Uṣaibiʿa with attribution to Īšōʿ bar ʿAlī.

Corroborative evidence for identifying the *Kunnāšā*’s author with Īšōʿ bar ʿAlī is provided by the only medical piece by him that has survived. Namely, manuscript Vat. sir. 217, ff. 226^v^–227^v^ contains a brief fragment reading as follows:^27^

ܬܘܒ ܟܬܒܝܢܢ ܩܠܝܠ ܡܢ ܟܬܒ ܪܒܢ ܝܫܘܥ ܒܪ ܥܠܝ ܬܠܡܝܕ ܪܒܢ ܚܘܢܝܢ ܪܝܫ ܐܣܘ̈ܬܐ. ܗ̇ܘ ܕܥܠ ܐܣܝܘܬܐ ܬܡܝܗܐܝܬ ܘܝܬܝܪܐ ܒܚܬܝܬܘܬܐ ܡܢ ܕܡܝܝ̈ܘܗܝ

We also write a fragment from the book on medicine of Rabban Īšōʿ bar ʿAlī, a disciple of Rabban Ḥunayn, the head of the physicians. [It is] admirable and more precise than the similar [works].

The text was first mentioned in the eighteenth-century catalogue description of the manuscript^28^ and since then it has turned up in scholarly publications but never received special interest.^29^ In fact, upon close reading it may even seem that the rubric refers to Īšōʿ bar ʿAlī’s medical work mistakenly, because the fragment is nothing more than an inventory of Syriac and Arabic equivalents for Greek drug names. The *Lexicon* of Īšōʿ bar ʿAlī could certainly fit well as a possible source of the fragment^30^ but examination brings negative results because, first of all, although the drug names do feature in the *Lexicon* they are dispersed among many other words, and secondly, the content of the entries is slightly different. With the availability of the new source, one may wonder whether the fragment was drawn from the *Kunnāšā*. Indeed, there is an exact correspondence between the inventory and the list of the simples present in the first Mēmrā. However the texts are not absolutely identical and in order to understand the difference, I would like to make a brief digression about the composition of the list of simples in the *Kunnāšā*.

While aiming to present the simples and their healing properties Īšōʿ bar ʿAlī could do no better than to exploit the relevant part of Galen’s *Simple Drugs* (to wit, books vi–viii). Considering the vast size of Galen’s text, the obvious solution was to make an abridgement that, however, retains the drugs’ original order following the Greek alphabet. Taking advantage of the available Syriac translation of the *Simple Drugs*, Īšōʿ bar ʿAlī added his own touch to the list. Namely, the Syriac version of the *Simple Drugs* must have already contained the Syriac equivalents of the Greek drug-names embedded in the text whereas the original Greek terms were reproduced in transliteration. Apparently, Īšōʿ bar ʿAlī was trying to keep up with developments in the field of medicine, particularly with the growing role of Arabic, and for that reason it is quite natural that he decided to increase the usefulness of the list by providing it with Arabic equivalents.^31^ Hence, as a rule we find both Syriac and Arabic equivalents for each Greek drug name.

Īšōʿ bar ʿAlī’s efforts to render Galen’s catalogue of drugs more practical and user-friendly were undoubtedly rewarded and the fragment in the Vatican manuscript offers good proof for that. We cannot be sure that there is a direct relationship between the *Kunnāšā* and the fragment, because the list covers only the drug names from *alpha* through *delta* and thus it is possible that it was copied not from the original treatise but from an intermediary version. Whatever the case may be, it is the actual content of the fragment that is worthy of notice. What we find in it is exclusively the Greek terms and their Syriac and Arabic equivalents.

To illustrate the situation let us look at the first three terms in Galen’s catalogue. Galen’s list begins with discussions of ἀβρότονον, ἄγνοϲ and ἄγρωστιϲ. The presentation of the drugs’ properties is quite lengthy and occupies eight, three and one pages in Kühn’s edition respectively.^32^ Īšōʿ bar ʿAlī masterfully condenses all that material into just a few lines (seven, four and three respectively), supplying the Greek terms with Syriac and Arabic equivalents (sop 238, f. 39^v^). The *Lexicon* and the Vatican fragment provide only the drugs’ names.

**Table:** 

L1 *ἀβρότονον* (*“wormwood,” Artemisia abrotanum*)	
*Lexicon* (Hoffmann, *Syrisch-arabische* *Glossen*, p. 9 no. 91)	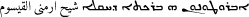
abrwṬwnwn—*brāktā d-ḥaqlā—šīḥ Armanī*^33^*—al-qayṣūm*^34^	
*Kunnāšā* (sop 238, f. 39^v^)	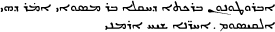
abrwṬwnwn—*brāktā d-ḥaqlā*, Bar Masawāi^35^ says that it is *al-qayṣūm*, other [say that it is] *šīḥ Armanī*.	
Vat. sir. 217, f. 226^v^	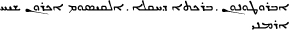
abrwṬwnwn—*brāktā d-ḥaqlā—al-qayṣūm*, other [say that it is] *šīḥ Armanī*.	
L2 *ἄγνοϲ* (*“chaste-tree,” Vitex agnus-castus*)	
*Lexicon* (Hoffmann, *Syrisch-arabische Glossen*, p. 11 no.181)	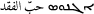
agnws—*ḥabb al-faqad*	
*Kunnāšā* (sop 238, f. 39^v^)	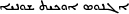
agnws that is *šunāyā*	
Vat. sir. 217, f. 226^v^	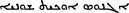
agnws that is *šunāyā*	
L3 *ἄγρωστιϲ* (*“dog’s tooth grass,” Cynodon dactylon*)	
*Lexicon* (Hoffmann, *Syrisch-arabische* *Glossen*, p. 12 no. 204)	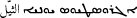
agrwsṬyws Greek *ṯayyīl*	
*Kunnāšā* (sop 238, f. 39^v^)	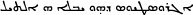
agrwsṬyws is *yablā* that is *ṯayyīl*	
Vat. sir. 217, f. 226^v^	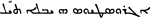
agrwsṬyws is *yablā ṯayyīl*.	

First of all, if we compare the *Lexicon* with the *Kunnāšā* we observe the following difference: The *Lexicon*’s entries are more condensed and omit the reference to the informants (L1); the Syriac equivalents feature more as an exception (L1), whereas normally we find only the Arabic ones (L2 and L3) that are the primary focus of the *Lexicon*. The *Kunnāšā*’s approach is different for it tends to provide both the Syriac and Arabic equivalent (with the exception of L2 where only the Syriac one is indicated). The same procedure is displayed by the Vatican fragment that indicates both the Syriac and Arabic equivalents (with the exception of L2). There thus can be no doubt that the Vatican fragment is based on the *Kunnāšā* and not on the *Lexicon*.

In terms of possible sources that supplied the necessary information, it can be noted that the Syriac equivalents in L1 and L3 were introduced already by Sergius of Rēšʿainā in his translation of the *Simple Drugs*.^36^ The Syriac term is followed by two Arabic equivalents, the first one provided on the authority of Bar Masawāi (Abū Zakarīyāʾ Yūḥannā ibn Māsawayh), whereas the source of the second equivalent is not identified. In the case of L2, Sergius of Rēšʿainā does not provide any equivalent here but the term *šunāyā* must have featured in Ḥunayn’s translation of the *Simple Drugs*.^37^

Having clarified the relationship between the *Kunnāšā* and the fragment preserved in the Vatican manuscript, we are justified in relying on the authorial attribution of the latter to Īšōʿ bar ʿAlī. After all, the extolling epithet (“admirable and thoroughly precise”) can by all means be applied to the text of the *Kunnāšā*.

It is worth noting in passing that the same Vatican manuscript also contains other fragments that match the text of the *Kunnāšā*. In particular, we find there (Vat sir. 217, ff. 221^v^–224^v^) yet another inventory, this time of simples that are classified according to their primary qualities (hot, cold, dry and wet). The classification is followed by a list of substitute drugs (Vat sir. 217, ff. 224^v^–226^r^). Both lists are present in the *Kunnāšā*. The classification of the simples is present in Mēmrā i.70–85 (sop 238, ff. 75^v^–77^v^) whereas the substitute drugs appear at the very end of the treatise, Mēmrā vii.25 (sop 238, ff. 429^r^–430^v^). This disconnected presence of the three fragments supports the assumption that they may not depend directly on the *Kunnāšā* but rather on an intermediary medical compilation that contained selected material from the *Kunnāšā*. That issue requires further study.^38^

## Conclusions

It is hard to overestimate the significance of a thirteenth-century medical manuscript preserved in the Syriac Orthodox Patriarchate. Even in its damaged form, the unique manuscript preserves the most extensive and voluminous Syriac medical treatise known to date and will undoubtedly change the entire field of Syriac medicine.^39^ In brief, the treatise shows that Syriac medicine at the peak of its development produced an elaborate system of the science of medicine that is intimately related to medieval medicine in both the Arabic and the Latin realms.^40^

A preliminary study of the text allows it to be identified as a medical handbook (*Kunnāšā*) that was written by Īšōʿ bar ʿAlī, who was a disciple of Ḥunayn b. Isḥāq, a personal physician of Caliph al-Muʿtamid (r. 870–892) and the author of the Syriac-Arabic *Lexicon*. This identification is supported by a fragment from the *Kunnāšā* preserved in a Vatican manuscript with an explicit attribution to Īšōʿ bar ʿAlī as well as by a reference in the *Kunnāšā* itself stating that its author also produced a treatise about poisons in two books. It is that treatise of Īšōʿ bar ʿAlī that was known in the Arabic medical tradition. A lack of references to the text in Arabic medical works is likely to be indicative of the fact that it was never translated into Arabic.^41^

In terms of its composition and content, the *Kunnāšā* appears to be greatly indebted to the *Pragmateia* of Paul of Aegina. Its impact can be traced both on the level of composition and in the discussion of individual ailments. Nevertheless, the author of the *Kunnāšā* was evidently compiling his handbook from multiple sources and his actual engagement with preceding traditions is yet to be determined. Īšōʿ bar ʿAlī’s personal input also deserves special attention for the apparent practical orientation of the *Kunnāšā* indicates that its author was a practitioner with deep expertise in maintaining health and treating diseases.

Being the product of a ninth-century scholar and intellectual who stood right at the centre of the burgeoning of Arabic medicine, the *Kunnāšā* grants us an unprecedented opportunity to scrutinize the state of contemporaneous Syriac medicine that was massively contributing to the formation and growth of the medical sciences in Arabic. Metaphorically speaking, the manual of Īšōʿ bar ʿAlī stands on the shoulders of the four-century long tradition of Syriac medicine that stems from late antique Galenism inherited from Alexandria. Although the works of Galen and the Alexandrian commentaries constituted the backbone of medicine as studied and practiced by the Syriac Christians, it was also indebted to Persian and Indian medical lore.^42^

In fact, in the case of the *Kunnāšā* we may even be dealing with one of the latest medical compositions in Syriac^43^ that postdates the extant medical works of Ḥunayn b. Isḥāq. It would therefore a fascinating task of its own to explore whether the author was able to produce a coherent medical system while being confronted with a massive corpus of medical literature that must have presented many conflicting theories and incompatible practical recommendations. In order to reveal the profile of the *Kunnāšā*, one may also compare it with roughly contemporary Arabic medical manuals, such as the *Paradise of Wisdom* (*Firdaws al-ḥikma*) of ʿAlī b. Rabban al-Ṭabarī and the *Book of Treasure* (*Kitāb al-Dhakhīra*).

Undoubtedly, the *Kunnāšā* will prove a document of major significance with regard to the availability of medical works in ninth-century Baghdad. Citations that range from Hippocrates to Ḥunayn show the *Kunnāšā* to be an indispensable witness to both Syriac translations of the classical Greek sources and indigenous Syriac medical works whose main body is lost.^44^ For instance, due to the direct practical significance of Galen’s catalogue of simples, numerous attempts were made to render the treatise first in Syriac and later in Arabic.^45^ The first Syriac translation was made by Sergius of Rēšʿainā, whereas the last one was perhaps implemented by Ḥunayn. Since the abridged version preserved in the *Kunnāšā* differs from Sergius’ translation it becomes an important witness for the later translation history of the *Simple Drugs* in the Syriac tradition.

A study of the *Kunnāšā* is, however, not lacking in serious difficulties, the least of which is a small tight handwriting that presents a challenge even to reading the text. The main obstacle is posed by the underdevelopment of the field of Syriac medicine and particularly by our meagre awareness concerning the translation techniques employed by the eighth- and ninth-century translators and the lexicography of such areas of medicine as anatomy, therapeutics, nosology and pharmacology.^46^ Unless considerable progress is achieved in those two directions, any study of the *Kunnāšā* will be uncritical and premature.

## Figures and Tables

**Figure:**
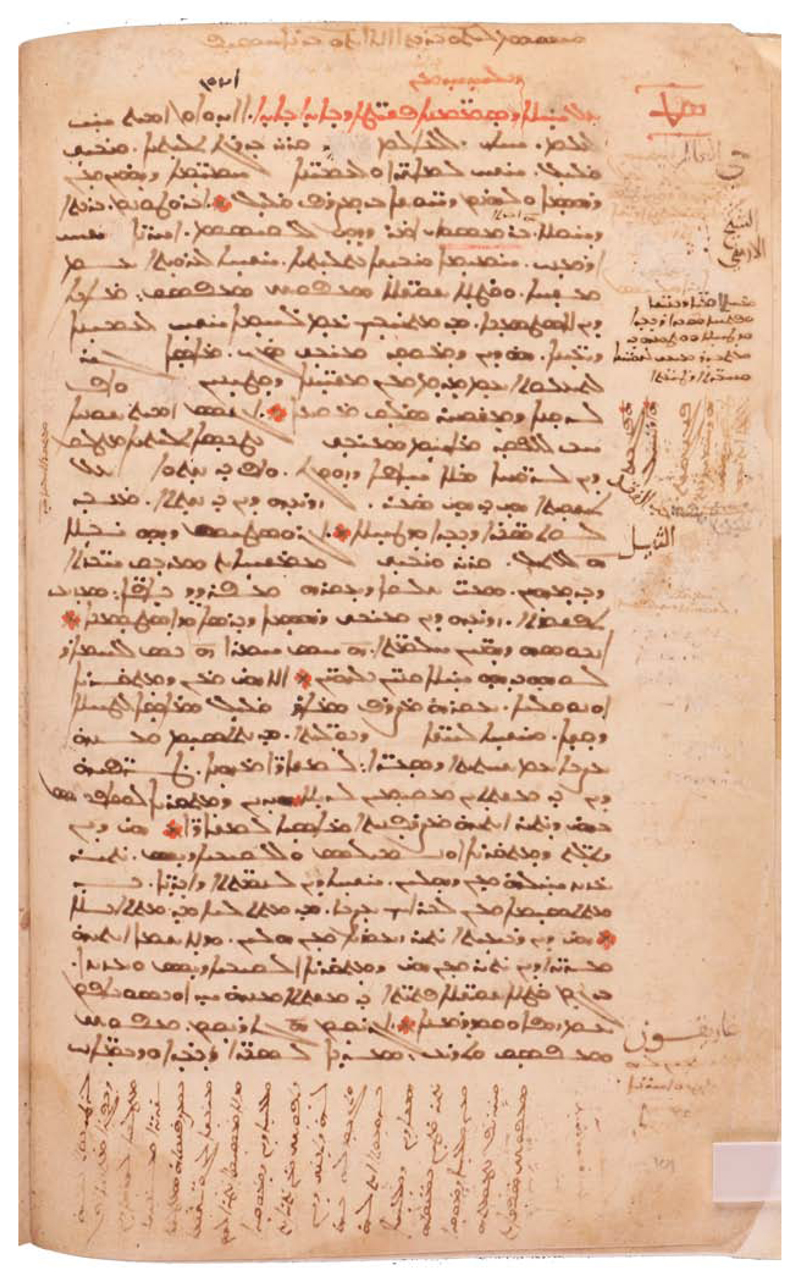
sop 238, f. 39^v^ (beginning of the inventory of the simples) Reproduced with kind permission of the Syriac Orthodox Patriarchate

## Footnotes

1 An unspecific catalogue description is available in Dōlabānī, “Catalogue des manuscrits,” p. 586. The manuscript was also mentioned in Barsoum, *The Scattered Pearls*, p. 187 (Barsoum attributes the work to Ḥunayn) and Brock, *The Hidden Pearl*, p. 161 and 133 (information is provided based on the catalogue description). The manuscript does not contain Ḥunayn’s treatise *Properties of Foodstuffs*, as assumed in Hawley, “Preliminary Notes,” p. 82 n. 9. To the best of my knowledge the manuscript was not photographed by Arthur Vööbus, who visited that collection and copied some manuscripts. The library of the Patriarchate of the Syriac Orthodox Church contains a few more medical manuscripts, one of which is the so-called *Syriac Epidemics* (Kessel, “The Syriac Epidemics”).

2 Mentioned also are Democritus and Philo of Tarsus.

3 Another scribal note can be found after Mēmrā vi (sop 238, f. 361^r^), where the scribe indicates the current date as July 16, ag 1529 [= 1218 ce].

4 One wonders if this is the same Abu Saʿid whose anatomical treatise survived in Armenian (edition: Vardanian, [*Abu Saʿid. On the Composition of Man*]). His floruit is however usually placed slightly earlier, in the second half of the twelfth century (Mahé, “La version arméni-enne”).

5 The scribe is mentioned in Barsoum, *The Scattered Pearls*, p. 534 but without additional information. See also Brock, *The Hidden Pearl*, p. 133. A partial facsimile edition of one of the modern copies along with English translation can be found in Mingana, *Encyclopædia*.

6 I hope to explore the issue on a different occasion. On Basil Meliteniotes see Korobeinikov, “A Greek Orthodox Armenian.”

7 The numeration of the chapters breaks off after chapter number 69.

8 Particularly mentioned are albanum (‮ܚܠܒܢܝܬܐ‬‎) and aloe (‮ܥܠܘܐ‬‎). A number of recipes are introduced as borrowed from the works of Paul [of Aegina] and Hermes.

9 On the genre see: Dietrich, “ʿAlī ibn Riḍwān,” Karmi, “A Mediaeval Compendium,” Meyerhof, “ʿAlî aṭ-Ṭabarī’s,” Meyerhof, “The *Book of Treasure*.”

10 The translation is that of Adams (Adams, *The Seven Books*, p. 383) with slight modifications.

11 The additions made by the author of the *Kunnāšā* are underlined.

12 Needs to be emended in ܥܠܒ.

13 Syriac *marqqānā* (hypochondriac) can be recognized in Arabic *marāqqīya* that features in the Arabic version of the text (see Pormann, *The Oriental Tradition*, pp. 78–79).

14 Peter E. Pormann kindly provided me with his draft edition of the chapter.

15 Pormann, *The Oriental Tradition*, pp. 13–46.

16 On the influence of the *Pragmateia* (mostly in the Arabic medical tradition) see Pormann, *The Oriental Tradition*, pp. 293–309.

17 The nature of the *Lexicon* of Īšōʿ bar ʿAlī suggests that we should rather place him in the area of Syro-Arabic translations. See also an attempt to attribute to him an Arabic translation of the *Diatessaron* from Syriac (Higgins, “The Arabic Version”), contrary to the widespread opinion that the translation was produced by his coreligionist Abū l-Faraǧ ʿAbd Allāh b. al-Ṭayyib whereas Īšōʿ bar ʿAlī was just the scribe of a Syriac copy of the *Diatessaron*. Higgins’ position met with strong disapproval (Baarda, “The Author,” Joosse, “An Introduction”).

18 Degen, “Zur syrischen Übersetzung.”

19 Overwien, “The Paradigmatic Translator,” Pormann and Joosse, “Commentaries,” Kessel, “*Life is Short, the Art is Long*,” Mimura, “Comparing interpretative glosses,” Pormann, “Al-Tarǧamāt al-Yūnānīya,” as well as the PhD of Samuel Barry, *The Question of Syriac Influence*.

20 Riad, *Studies*.

21 Edition: Hoffmann, *Syrisch-arabische Glossen*, Gottheil, *Bar ʿAlī (Īshōʿ)*. On the genre of bilingual dictionaries see Endress, “Bilingual lexical materials.” We find *mappaq b-rūḥā* also in Īšōʿ bar ʿAlī’s *Lexicon* (edition: Hoffmann, *Syrisch-arabische Glossen*, pp. 1–5; cf. Riad, *Studies*, p. 35).

22 Butts, “The Biography,” p. 70.

23 Ullmann, *Medizin*, pp. 208–209, Sezgin, *Geschichte*, pp. 337–340.

24 See, most recently, Debié, “Sciences,” p. 14.

25 Sezgin, *Geschichte*, p. 377. See a brief presentation of the text in Raggetti, “The ‘Science of Properties’ and its Transmission,” pp. 169–171 and Contadini, *A World of Beasts*, p. 8. A critical edition of the treatise is under preparation by Lucia Raggetti (Freie Universität, Berlin).

26 Ullmann, *Medizin*, p. 327, Sezgin, *Geschichte*, p. 259.

27 Butts erroneously reads ‮ܐܣܝ̈ܘܬܐ‬‎ instead of ‮ܐܣܘ̈ܬܐ‬‎ (Butts, “The Biography,” p. 68).

28 The manuscript does not contain a colophon but can be dated to the 16th c. Description: Assemanus, *Bibliothecæ Apostolicæ Vaticanæ*, pp. 503–505.

29 Baumstark, *Geschichte der syrischen Literatur*, p. 241 n. 16, Butts, “The Biography,” p. 68.

30 This is how the fragment was identified by Butts who however does not provide an exact correspondence between two texts (Butts, “The Biography,” p. 68). For a similar lexicographical interpretation of the text see Graf, *Geschichte*, p. 157.

31 It would however be too hasty to attribute the authorship of the Arabic equivalents to Īšōʿ bar ʿAlī. Rather, it is more likely that he was following the standard Arabic pharmacological nomenclature of his time. Besides translations of pharmacological treatises, there existed also inventories of (not only medical) synonyms (cf. Ullmann, *Medizin*, pp. 288–292).

32 Kühn, *Claudii Galeni Opera*, pp. 798–811.

33 “Armenian wormwood,” *Artemisia armeniaca*.

34 “Southern-wood,” *Artemisia abrotanum*.

35 Abū Zakarīyāʾ Yūḥannā b. Māsawayh (d. 857) (Ullmann, *Medizin*, pp. 112–115). Although it is hardly possible to identify the work used by Īšōʿ bar ʿAlī it is worth noting that a number of references to Ibn Māsawayh on wormwood are preserved in al-Bīrūnī’s pharmacological treatise *Kitāb al-Ṣaydana*.

36 Merx, “Proben der syrischen Übersetzung,” p. 249. On that trait of Sergius’ translation see Bhayro, “Syriac Medical Terminology,” pp. 162–165, and Bhayro and Hawley, “La littérature botanique,” pp. 296–297.

37 At least the term is present in Ḥunayn’s treatise *Properties of Foodstuffs* (Hawley, “Preliminary Notes,” p. 91, entry number ii.84).

38 The same Vatican manuscript also contains one medical fragment (Vat. sir. 217, ff. 220^r^–221^v^) that deals with the four faculties (attractive, retentive, alterative and expulsive). However, I was unable to find a corresponding place in the *Kunnāšā*. Curiously enough, that entire section—consisting of an unidentified fragment, classification of simples and the inventory of substitute drugs (Vat sir. 217, ff. 220^r^–226^r^)—is attributed in the catalogue to Ḥunayn b. Isḥāq (Assemanus, *Bibliothecæ Apostolicæ Vaticanæ*, p. 504 and repeated also in Vööbus, “Discoveries,” p. 528), although there is absolutely no indication of this in the manuscript (noticed already in Degen, “Ein Corpus,” p. 121 n. 41).

39 The text is roughly three times larger than the therapeutical part of the *Syriac Book of Medicines*.

40 And as portrayed, for example, Porman and Savage-Smith, *Medieval Islamic Medicine*, pp. 41–67, and in greater detail in Demaitre, *Medieval Medicine*.

41 See, for example, besides the standard works of Sezgin (Sezgin, *Geschichte*) and Ullmann (Ullmann, *Medizin*) a recent study on the tenth century medical compendium of Abū al-Ḥasan Aḥmad b. Muḥammad aṭ-Ṭabarī (Ambjörn, “Book-Titles”).

42 For a general survey of Syriac medicine see my “Syriac Medicine.”

43 If we exclude the elusive medical works of Bar ʿEbrōyō (d. 1286), at least the predominant part of which, if not all, was composed in Arabic (Micheau, “Les traités médicaux,” Joosse, “A Newly-Discovered Commentary”).

44 For a survey of Syriac medical literature see Habbi, “Textes médicaux”. Some Syriac medical works are known to us thanks to the quotations preserved in Arabic medical treatises. The largest number of excerpts can be found in the *Comprehensive Book* (*al-Kitāb al-Ḥāwī*) of Abū Bakr Muḥammad ibn Zakarīyāʾ al-Rāzī (d. 925 or 935) and that have recently become conveniently available in English translation (Kahl, *The Sanskrit, Syriac and Persian Sources*, pp. 160–364).

45 Pormann, “The Development.”

46 Syriac pharmacology has recently received a welcome tool (Gignoux, *Lexique*) that is however based on just a limited number of sources. I should also mention that a number of projects are underway that are going to contribute to the progress of Syriac medicine (for example, Robert Hawley’s edition of the Syriac and Arabic versions of Ḥunayn’s treatise on *Properties of Foodstuffs* and Sergius’ translation of Books vi–viii of Galen’s *Simple Drugs*). For challenges that one has to face while dealing with semantic ambiguity and overlap in medical terminology see, for instance, Ford, “Two Syriac Terms”. Similar problems pertain also to Graeco-Arabic studies (Pormann, *The Oriental Tradition*, 123–288 and Pormann, “The Formation”).

## References

[R1] Adams Francis (1844). The Seven Books of Paulus of Ægineta.

[R2] Ambjörn Lena (2011). Book-Titles mentioned in the 10th Century Medical Encyclopedia *Al-muʿālajāt al-buqrāṭiyya*. Galenos.

[R3] Assemanus Stephanus Evodius, Simonius Joseph (1759). Bibliothecæ Apostolicæ Vaticanæ codicum manuscriptorum catalogus.

[R4] Baarda Tjitze, Baarda Tjitze (1978). The Author of the Arabic Diatessaron. Miscellanea Neotestamentica.

[R5] Barry Samuel Chew (2016). The Question of Syriac Influece Upon Early Arabic Translation of the Aphorisms of Hippocrates.

[R6] Barsoum Ignatius Aphram, Moosa Matti (2003). The Scattered Pearls. A History of Syriac Literature and Sciences.

[R7] Baumstark Anton (1922). Geschichte der syrischen Literatur mit Ausschluß der christlich-palästinensischen Texte.

[R8] Bhayro Siam (2005). Syriac Medical Terminology: Sergius and Galen’s Pharmacopia. Aramaic Studies.

[R9] Bhayro Siam, Robert Hawley, Villey Émilie (2014). La littérature botanique et pharmaceutique en langue syriaque. Les sciences en syriaque.

[R10] Brock Sebastian P, Taylor David GK (2001). The Hidden Pearl.

[R11] Butts Aaron Michael (2009). The Biography of the Lexicographer Ishoʿ bar ʿAli (ʿĪsā b. ʿAlī). Oriens Christianus.

[R12] Contadini Anna (2011). A World of Beasts: A Thirteenth-Century Illustrated Arabic Book on Animals (the Kitāb Naʿt al-Ḥayawān) in the Ibn Bakhtīshūʿ tradition.

[R13] Debié Muriél, Villey Émilie (2014). Sciences et savants syriaques: une histoire multiculturelle. Les sciences en syriaque.

[R14] Degen Rainer (1972). Ein Corpus Medicorum Syriacorum. Medizinhistorisches Journal.

[R15] Degen Rainer (1978). Zur syrischen Übersetzung der Aphorismen des Hippokrates. Oriens Christianus.

[R16] Demaitre Luke (2013). Medieval Medicine. The Art of Healing, from Head to Toe.

[R17] Dietrich Albrecht, Gabrieli Francesco, Traini Renato (1984). ʿAlī ibn Riḍwān über den Wert medizinischer Lehrbücher (Kanānīš). Studi in onore di Francesco Gabrieli nel suo ottantesimo compleanno.

[R18] Dōlabānī Yūḥannā, René Lavenant, Sebastian Brock, Khalil Samir (1994). Catalogue des manuscrits de la bibliothèque du patriarcat syrien orthodoxe à Homs (auj. à Damas). Parole de l’Orient.

[R19] Endress Gerhardt, Hamesse Jacqueline, Jacquart Danielle (2001). Bilingual lexical materials in the Arabic tradition of the Hellenistic sciences. Lexiques bilingues dans les domaines philosophique et scientifique (Moyen Âge—Renaissance).

[R20] Ford James Nathan (2002). Two Syriac Terms Relating to Ophthalmology and their Cognates. Journal of Semitic Studies.

[R21] Gignoux Philippe (2011). Lexique des termes de la pharmacopée syriaque.

[R22] Gottheil Richard James Horatio (1910). Bar ʿAlī (Īshōʿ). The Syriac-Arabic Glosses.

[R23] Graf Georg (1947). Geschichte der christlichen arabischen Literatur.

[R24] Habbi Joseph, Fiaccadori Gianfranco (1990). Textes médicaux grecs en syriaque. Autori classici in lingue del Vicino e Medio Oriente.

[R25] Hawley Robert (2008). Preliminary Notes on a Syriac Treatise about the Medicinal Properties of Foodstuffs. Semitica et Classica.

[R26] Higgins Angus John Brockhurst (1944). The Arabic Version of Tatian’s Diatessaron. Journal of Theological Studies.

[R27] Hoffmann Georg (1874). Syrisch-arabische Glossen. Erster Band. Autographie einer Gothaischen Handschrift enthaltend Bar Ali’s Lexikon von Alaf bis Mim.

[R28] Joosse N Peter (1999). An Introduction to the Arabic Diatessaron. Oriens Christianus.

[R29] Joosse N Peter (2013). A Newly-Discovered Commentary on the Hippocratic Prognostic by Barhebraeus: Its Contents and Its Place within the Arabic *Taqdimat al-maʿrifa* Tradition. Oriens.

[R30] Karmi Ghada (1978). A Mediaeval Compendium of Arabic Medicine: Abū Sahl al-Masīḥī’s ‘Book of the Hundred’. Journal for the History of Arabic Science.

[R31] Kahl Oliver (2015). The Sanskrit, Syriac and Persian Sources in the Comprehensive Book of Rhazes.

[R32] Kessel Grigory, Peter E Pormann (2012). The Syriac Epidemics and the Problem of Its Identification. Epidemics in Context. Greek Commentaries on Hippocrates in the Arabic Tradition.

[R33] Kessel Grigory (2015). *Life is Short, the Art is Long*: An Interpretation of the First Hippocratic Aphorism by an East Syriac Monk in the 7th Century Iraq (Isaac of Nineveh, *Kephalaia gnostica* 3, 62). Zeitschrift für Antikes Christentum.

[R34] Kessel Grigory, Daniel King Syriac Medicine. The Syriac World.

[R35] Korobeinikov Dimitri, Rustam Shukurov (2009). A Greek Orthodox Armenian in the Seljukid Service: the Colophon of Basil Meliteniotes. Mare et litora. Essays presented to Sergei Karpov for his 60th birthday.

[R36] Kühn Karl Gottlob (1826). Claudii Galeni Opera omnia.

[R37] Mahé Jean-Pierre (2006). La version arménienne du médecin Abou-Saïd. Comptes rendus des séances de l’Académie des Inscriptions et Belles-Lettres.

[R38] Merx Adalbert (1885). Proben der syrischen Übersetzung von Galenus’ Schrift über die einfachen Heilmittel. Zeitschrift der Deutschen Morgenländischen Gesellschaft.

[R39] Meyerhof Max (1930). The *Book of Treasure* an early Arabic Treatise on Medicine. Isis.

[R40] Meyerhof Max (1931). Alî aṭ-Ṭabarī’s ‘Paradise of Wisdom’, one of the oldest Arabic Compendiums of Medicine. Isis.

[R41] Micheau Françoise (2008). Les traités médicaux de Barhebraeus. Parole de l’Orient.

[R42] Mimura Taro Comparing interpretative glosses in the Syriac and Arabic translations of the Hippocratic Aphorisms. Aramaic Studies.

[R43] Mingana Alphonse (1935). Encyclopædia of Philosophical and Natural Sciences as Taught in Baghdad about a.d. 817, or Book of Treasures by Job of Edessa.

[R44] Overwien Oliver (2015). The Paradigmatic Translator and His Method: Ḥunayn ibn Isḥāq’s Translation of the Hippocratic Aphorisms from Greek via Syriac into Arabic. Intellectual History of the Islamicate World.

[R45] Pognon Henri (1903). Une version syriaque des aphorismes d’Hippocrate.

[R46] Pormann Peter Ernst (2004). The Oriental Tradition of Paul of Aegina’s ‘Pragmateia’.

[R47] Pormann Peter Ernst (2011). The Formation of the Arabic Pharmacology: Between Tradition and Innovation. Annals of Science.

[R48] Pormann Peter Ernst, Hansberger Rotraud, Afifi al-Akiti Muhammad, Burnett Charles (2012). The Development of Translation Techniques from Greek into Syriac and Arabic: The Case of Galen’s On the Faculties and Powers of Simple Drugs, Book Six. Medieval Arabic Thought.

[R49] Pormann Peter Ernst, Koetschet Pauline, Pormann Peter E (2016). Al-Tarǧamāt al-Yūnānīya al-Suryānīya al-ʿArabīya li-l-nuṣūṣ al-ṭibbīya fī awāʾil al-ʿaṣr al-ʿAbbāsī. Našʾat al-Ṭibb al-ʿArabī fī l-qurūn al-wusṭā (La construction de la médecine arabe médiévale).

[R50] Pormann Peter Ernst, Joosse N Peter, Pormann Peter E (2012). Commentaries on the Hippocratic Aphorisms in the Arabic Tradition. Epidemics in Context.

[R51] Pormann Peter Ernst, Savage-Smith Emilie (2007). Medieval Islamic medicine.

[R52] Raggetti Lucia, Johnson J Cale (2015). The ‘Science of Properties’ and its Transmission. In the Wake of the Compendia: Infrastructural Contexts and the Licensing of Empiricism in Ancient and Medieval Mesopotamia.

[R53] Riad Eva (1988). Studies in the Syriac Preface.

[R54] Sezgin Fuat (1970). Geschichte des arabischen Schrifttums.

[R55] Ullmann Manfred (1970). Medizin im Islam.

[R56] Vardanian Stella (1974). [Abu Saʿid. On the Composition of Man].

[R57] Vööbus Arthur (1974). Discoveries of New Syriac Manuscripts on Hunain. Ephram Hunayn Festival.

